# What makes a life event traumatic for a child? The predictive values of DSM-Criteria A1 and A2

**DOI:** 10.3402/ejpt.v4i0.20436

**Published:** 2013-08-21

**Authors:** Eva Verlinden, Mirjam Schippers, Els P. M. Van Meijel, Renée Beer, Brent C. Opmeer, Miranda Olff, Frits Boer, Ramón J. L. Lindauer

**Affiliations:** 1 Department of Child and Adolescent Psychiatry, Academic Medical Center, University of Amsterdam, Amsterdam, the Netherlands; 2 De Bascule, Academic Center for Child and Adolescent Psychiatry, Amsterdam, the Netherlands; 3 Clinical Research Unit, Academic Medical Center, University of Amsterdam, Amsterdam, the Netherlands; 4 Department of Psychiatry, Academic Medical Center, University of Amsterdam, Amsterdam, the Netherlands

**Keywords:** posttraumatic stress disorder (PTSD), trauma, diagnosis, stressor criterion, Diagnostic and Statistical Manual, classification, assessment, DSM-5

## Abstract

**Background:**

The *Diagnostic and Statistical Manual of Mental Disorders* (DSM)-Criteria A1 and A2 for posttraumatic stress disorder (PTSD) have been discussed extensively, with several studies in adults or adolescents supporting the removal of Criterion A2. However, solid research in children is missing.

**Objective:**

This study evaluated the DSM-Criteria A1 and A2 in predicting posttraumatic stress in children.

**Method:**

A sample of 588 Dutch school children, aged 8–18 years, completed a self-report questionnaire to determine if they met Criteria A1 and/or A2. Their posttraumatic stress response was assessed using the Children's Revised Impact of Event Scale.

**Results:**

The contribution of Criterion A2 to the prediction of posttraumatic stress in children is of greater importance than the contribution of Criterion A1. Children who met Criterion A2 reported significantly higher levels of posttraumatic stress and were nine times more likely to develop probable PTSD than children who did not meet Criterion A2. When Criterion A1 was met, a child was only two times more likely to develop probable PTSD as compared with those where Criterion A1 was not met. Furthermore, the low sensitivity of Criterion A1 suggests that children may regularly develop severe posttraumatic stress in the absence of Criterion A1. The remarkably high negative predictive value of Criterion A2 indicates that if a child does not have a subjective reaction during an event that it is unlikely that he or she will develop PTSD.

**Conclusions:**

In contrast to most adult studies, the findings of this study emphasize the significant contribution of Criterion A2 to the prediction of posttraumatic stress in children and raise fundamental questions about the value of the current Criterion A1.

Over the past two decades, epidemiologic research on child traumatic stress has demonstrated that children's exposure to traumatic events is more common than once thought (Costello, Erkanli, Fairbank, & Angold, 2002; Fairbank & Fairbank, 2009). Furthermore, children exposed to traumatic events are at high risk for developing a wide range of mental and physical health problems, including posttraumatic stress disorder (PTSD; De Bellis & Van Dillen, 2005; Kearney, Wechsler, Kaur, & Lemos-Miller, 2010). If we can predict which children are at risk of developing PTSD, then interventions can be started early. However, the definition of PTSD has been controversial since its introduction in the *Diagnostic and Statistical Manual of Mental Disorders*, Third Edition (DSM-III; American Psychiatric Association, 1980). One controversy regards the definition of criteria that need to be fulfilled to consider an event traumatic. These uncertainties in definition preclude an accurate prediction of PTSD.

The DSM criterion defining which events qualify as traumatic is known as Criterion A. This criterion has been discussed extensively and has changed in each version of the DSM (Weathers & Keane, 2007). According to the current DSM-IV-TR (American Psychiatric Association, 2000, p. 467), Criterion A contains a two-folded definition of the traumatic event. The first refers to an objective level of severity, known as Criterion A1. As stated in the DSM-IV-TR: “The person experienced, witnessed, or was confronted with an event or events that involved actual or threatened death or serious injury, or a threat to the physical integrity of oneself or others.” The second, known as Criterion A2, refers to a subjective level of severity: “The person's response to the event must involve intense fear, helplessness, or horror.” Both Criteria A1 and A2 must be met in order to qualify an experienced event as traumatic. This implies that it is not possible to diagnose PTSD in the absence of one of these requirements, even if all other symptoms are met. Therefore, by using the current DSM-IV-TR definition of PTSD, there might be children who are clearly symptomatic and impaired but do not fulfill Criterion A1 or A2 for the actual diagnosis of PTSD. This may exclude them from receiving proper trauma-focused treatment.

The upcoming DSM-5 inspires researchers to present recommendations on the PTSD diagnosis, aiming for a more accurate PTSD definition. Preliminary draft revisions that have been proposed by the DSM-5 Work Groups suggest to retain Criterion A1 and remove Criterion A2. The main reason proposed for retaining Criterion A1 is that in most cases PTSD does not develop unless a person experienced an event that is extremely stressful (Friedman, Resick, Bryant, & Brewin, 2011). The rationale to eliminate Criterion A2 is that it is considered to lack any added value. Research by Bedard-Gilligan and Zoellner (2008) showed that Criterion A2 added little to the ability of Criterion A1 to predict PTSD symptoms. They found that only the absence of Criterion A2 predicted the absence of PTSD symptoms. Furthermore, Criterion A2 appears to be of limited value when applied to individuals who are trained to handle occupationally related traumatic events, such as military personnel and police officers (Adler, Wright, Bliese, Eckford, & Hoge, 2008; Friedman et al., 2011). These individuals may not respond to a traumatic event with fear, helplessness, or horror, because of their professional training.

The above revisions, however, are mainly based on research in adults and adolescents. Children experience events differently in comparison to adults. Because of their lack of experience in the world, children may perceive some events as extremely stressful, which most adults would not, or vice versa. Furthermore, whether an event should be considered overwhelming depends upon the developmental capacities of a child (Scheeringa & Gaensbauer, 2000). For these reasons, it seems to be difficult to objectify Criterion A1 in children. In addition, there are indications that children's subjective experiences of events (Criterion A2) might play a crucial role in the development of PTSD. In children exposed to an earthquake, for example, Giannopoulou et al. (2006) found that the severity of symptoms was strongly predicted by perceived threat. Similar results were found in a prospective study with children who experienced a road traffic accident (Stallard, Velleman, & Baldwin, 1998). This study showed that the presence of PTSD was not related to the objective nature of the accident or the injuries, but was significantly associated with the subjective appraisal of threat to life. In addition, Blom and Oberink (2012) critically reviewed the validity of the DSM-IV PTSD criteria in children and adolescents exposed to traumatic events. They suggested that, according to the majority of empirical findings, the emotional reactions are predictive of PTSD in children. Taken together, the objective Criterion A1 may be insufficient to explain the development of PTSD in children. Rather, an event must be subjectively experienced as traumatic before a child is likely to develop PTSD (Creamer, McFarlane, & Burgess, 2005; Friedman et al., 2011).

Supported by studies mainly in adults or adolescents, Criterion A2 will most likely be removed in the upcoming DSM-5. However, solid research in children is still missing. Therefore, the purpose of this study was to examine the predictive values of both DSM-Criteria A1 and A2 in children. We addressed the following research question: what are the contributions of Criterion A1 and Criterion A2 to the prediction of the posttraumatic stress response in children? The results of this study will contribute to the development of an accurate PTSD definition in the upcoming DSM-5.

## Method

### Design

This cross-sectional study comprises a sample of 643 school children, aged 8–18 years, recruited from two primary schools (grades 5–8) and a large middle-class secondary school (middle-level education) in the Netherlands. Schools were selected based on their postal code, region, and level of education in order to ensure that the participants in this study constitute a representative sample of school children regarding their socio-economic status (SES). Data were collected in January and March 2010 through a self-report questionnaire.

### Recruitment of participants

Prior to the study, approval was obtained from the Medical Ethical Committee of the Academic Medical Center in Amsterdam. Parents with children in primary school (8–11 years old) were informed about the study through a letter and were asked to sign informed consent (opting-in procedure). Parents with children in secondary school (12 years and above) were informed through a school letter. They had the possibility to inform the school if they did not want their child to participate (opting-out procedure). Children were informed about the study during class and were invited to participate voluntarily. In addition, all children in secondary school had to sign informed consent before participating.

### Measurements

In addition to demographic characteristics (including age, gender, and grade level), data on Criterion A and the posttraumatic stress response were obtained.

#### Criterion A

Self-report questions were designed specifically to assess Criteria A1 and A2 according to the DSM-IV-TR. First, to assess Criterion A1, children were asked to report the worst event they had ever experienced in response to an open-ended question (examples were given, such as a car accident, bullying, parental divorce, or violence). Children were asked to provide a full description of the event in a way that the researcher would understand what happened. This event was the focus for the following questions, that is, how long ago it took place and the number of times it occurred. Whether an event met Criterion A1 was determined by four different raters according to the majority scoring method (see “Procedure” section). Second, Criterion A2 was assessed. Children were asked to rate how they felt during or immediately after the described event in terms of fear, anger, sadness, helplessness, shame, guilt, and horror. Children responded on a Likert-style scale ranging from 0 (“not at all”) to 5 (“extremely”) for each feeling. Criterion A2 was coded positive if a child reported a score of 3 or higher on fear, helplessness, or horror.

#### Posttraumatic stress response

The posttraumatic stress response was assessed using the Dutch version of the Children's Revised Impact of Event Scale (CRIES-13; Olff, 2005). This measure is an adaptation of the Impact of Event Scale (IES; Horowitz, Wilner, & Alvarez, 1979), which was originally designed for adults. The CRIES-13 is a 13-item, self-report questionnaire designed to screen children for PTSD. It consists of four questions to assess intrusion, four questions to assess avoidance, and five questions to assess arousal. Each question is answered on a 4-point Likert-style scale (“not at all”=0; “rarely”=1; “sometimes”=3; and “often”=5). The total score indicates the severity of posttraumatic stress response and ranges from 0 to 65 (Giannopoulou et al., 2006). In order to classify children into two groups, a cutoff score of 30 or higher on the total score is used to indicate “probable PTSD” (Perrin, Meiser-Stedman, & Smith, 2005; Verlinden et al., 2013). Furthermore, children need to have at least one positive score (“sometimes” or “often”) within each of the symptom clusters (intrusion, avoidance, and arousal) in order to be classified with “probable PTSD.” The CRIES-13 has been successfully used in a number of studies with children aged 8 years and above (Children and War Foundation, 1998). Psychometric properties have been previously reported (Giannopoulou et al., 2006; Perrin et al., 2005; Smith, Perrin, & Dyregrov, 2003; Verlinden et al., 2013), showing the CRIES-13 to be a valid measure of posttraumatic stress. In this study, Cronbach's alpha for internal consistency was 0.84 for all items. Regarding the three different subscales, intrusion, avoidance, and arousal Cronbach's alpha was 0.77, 0.72, and 0.71, respectively.

### Procedure

Children filled out the questionnaire during class. The teacher and at least one researcher were present to answer any questions when necessary and to encourage children to give a full description of the worst event they had ever experienced. In addition, all children and teachers were well informed about the possibilities to (anonymously) contact the researchers or the Department of Child and Adolescent Psychiatry after participation in the study, if they had any further questions or concerns about the study or themselves. Demographic characteristics and questions concerning Criterion A were administered first, followed by the CRIES-13. Children were instructed to complete the CRIES-13 in reference to the event they had described before as the worst event.

Four raters determined independently whether the described events fulfilled Criterion A1 according to the DSM-IV-TR. The four raters included a research psychologist, a child psychiatrist, a clinical psychologist, and a professor in child psychiatry, all with extensive experience in the trauma field. The majority scoring method (Hovens & Van der Ploeg, 1993; Van Hooff, McFarlane, Baur, Abraham, & Barnes, 2009) was used to finally classify an event as a Criterion A1 event or not. In other words, an event was coded positive if at least three out of four raters nominated the event as fulfilling Criterion A1. An event was coded negative if at least three out of four raters nominated the event as not fulfilling Criterion A1. If there was no majority, but the raters were equally divided in their opinion, events were coded as equivocal. Events classified as equivocal were excluded for further analyses because it was unclear whether they should be classified as Criterion A1 or not.

### Statistical analyses

For the CRIES-13, data were counted as missing if more than one item on a subscale (>25%) was missing. Where only one item was missing on a subscale, this item was scored zero, and data were included (Smith, Perrin, Yule, Hacam, & Stuvland, 2002).

Sensitivity, specificity, and positive/negative predictive values were calculated to examine the predictive accuracy of Criteria A1 and A2.

The contribution of Criteria A1 and A2 to the prediction of posttraumatic stress symptoms was evaluated with a multiple linear regression analysis. Subsequently, a multiple logistic regression analysis was performed to evaluate the contribution of Criteria A1 and A2 to the prediction of probable PTSD.

Statistical significance was established at an alpha level of 0.05. Statistical analyses were conducted using Statistical Product and Service Solutions (SPSS) version 19 for Windows.

## Results

### Sample characteristics

A total of 643 children were present on the day of data collection, of which 588 children/parents (91%) provided informed consent. Of those who were willing to participate, 55 children did not report their worst event and were excluded. Therefore, the final sample after the *a priori* data exclusions consisted of 533 children. The sample had slightly more girls (56%) than boys with an average age of 13.6 years (SD=1.9). Most children were in secondary school (87%). Demographic characteristics are shown in Table 1.

**Table 1:** Demographic characteristics

Variable	M	SD	*n*	%
Age	13.6	1.9		
Sex				
Male			232	44
Female			298	56
Type of education				
Primary school			70	13
Secondary school—basic profession-oriented learning path			124	23
Secondary school—middle management-oriented learning path			184	35
Secondary school—mixed learning path			139	26
Secondary school—theoretical learning path			14	3

Note: Three children have not indicated gender and two children have not indicated the type of education. Basic profession-oriented learning path emphasizes vocational training. Middle management-oriented learning path is composed of an equal amount of theoretical education and vocational training. Mixed learning path emphasizes theoretical education, but still contains some amount of vocational training. Theoretical learning path has the largest share of theoretical education.

### Experienced events

Events brought up by the children were divided into nine broad categories (see Table 2). A tenth category named “other events” remained for the events that were reported by just one or two children and could not be placed in one of the other nine categories. “Death of a loved one” was mentioned most often as the worst event (40%) followed by “lost pet” (11%) and “bullying” (10%). About 20% of the events took place in the past year. Most events took place more than a year ago (48%) or more than 5 years ago (32%).

**Table 2:** Number of events classified as Criterion A1 and Criterion A2

	Criterion A1						Criterion A2			
		
	Yes		No		Equivocal		Yes		No	
					
Type of event	*n*	%	*n*	%	*n*	%	*n*	%	*n*	%
Death of a loved one	31	14.6	162	76.1	20	9.4	98	46.9	111	53.1
Lost pet	–	–	59	100	–	–	34	59.6	23	40.4
Bullying	4	7.4	46	85.2	4	7.4	41	77.4	12	22.6
Accident	44	86.3	3	5.9	4	7.8	26	53.1	23	46.9
Illness	10	27.8	19	52.8	7	19.4	23	69.7	10	30.3
Divorce of parents	–	–	30	93.8	2	6.3	14	45.2	17	54.8
Domestic violence	11	40.7	14	51.9	2	7.4	19	76	6	24.0
Sexual assault	12	92.3	–	–	1	7.7	13	100	–	–
Physical or verbal violence	11	100	–	–	–	–	10	90.9	1	9.1
Other events	6	16.2	31	83.8	–	–	26	72.2	10	27.8
Total	129	24.2	364	68.3	40	7.5	304	58.8	213	41.2

According to the majority scoring method, 24% of the events mentioned by the children were classified as Criterion A1 events and almost 59% of the events met Criterion A2 (see Table 2). Events classified as equivocal (*n*=40) were excluded from subsequent analyses because it was unclear whether to classify them as Criterion A1 or not. Missing data on Criterion A2 resulted in the exclusion of another 15 cases. Furthermore, 5 children were excluded due to more than one missing item on a subscale of the CRIES-13, leaving a total of 473 cases for further analyses. Twenty children had only one missing item on a subscale, these items were scored zero. Mean and standard deviations regarding the scores on the CRIES-13 based on the presence of Criteria A1 and/or A2 are shown in Table 3.

**Table 3:** Mean and standard deviations regarding scores on the CRIES-13 based on the presence of Criteria A1 and/or A2

	Total (*N*=473)		No A1/A2 (*n*=163)		Only A1 (*n*=32)		Only A2 (*n*=186)		Both A1 andA2 (*n*=92)	
					
Scale	M	SD	M	SD	M	SD	M	SD	M	SD
Intrusion	7.6	5.4	5.1	4.5	5.5	4.6	8.8	5.3	10.3	5.1
Avoidance	7.7	5.7	5.4	4.8	5.6	4.9	8.6	5.3	10.5	6.3
Hyperarousal	6.7	5.7	3.9	3.7	5.0	4.1	8.3	5.6	9.2	6.8
Total	22.0	13.6	14.5	9.7	16.0	10.4	25.7	12.5	30.0	15.3

### Prediction of posttraumatic stress

To examine the predictive accuracy of Criteria A1 and A2, sensitivity, specificity, and positive/negative predictive value were calculated. The results are shown in Table 4. Criterion A2 had high sensitivity, whereas sensitivity of Criterion A1 was quite low. Furthermore, Criterion A2 had strong negative predictive value.

**Table 4:** Numbers of probable PTSD and the related sensitivity, specificity, and predictive values

	Probable PTSD (*n*)						
					
Variable	Yes	No	Total	Sensitivity	Specificity	PPV	NPV
Criterion A1							
Yes	48	76	124	0.38	0.78	0.39	0.78
No	78	271	349				
							
Criterion A2							
Yes	113	165	278	0.90	0.52	0.41	0.93
No	13	182	195				
							
Criteria A1 and A2							
Yes	44	48	92	0.35	0.86	0.48	0.78
No	82	299	381				
Total	126	347	473				

Note: PPV, positive predictive value; NPV, negative predictive value.

Before conducting further analyses, preliminary analyses were conducted to evaluate potential collinearity problems. The tolerance value of Criteria A1 and A2 was 0.97 and the variance inflation factor (VIF) was 1.04. The tolerance value was >0.10; therefore, we have not violated the multicollinearity assumption. This is further supported by the VIF value, well below the commonly used cut-off of 10.

With multiple linear regression analysis, we then evaluated the contributions of Criteria A1 and A2 to the prediction of posttraumatic stress in children according to the total score on the CRIES-13. The linear combination of A1 and A2 was significantly related to the posttraumatic stress response, *F*(2,470)=64.0 (*p*<0.001). The multiple correlation coefficient was 0.46, indicating that approximately 21% of the variance of the posttraumatic stress response can be accounted for by the linear combination of Criteria A1 and A2. Criterion A1 alone accounted for only 4% of the variance of the posttraumatic stress response, while Criterion A2 contributed an additional 17%. On average, when Criterion A1 was met, children scored 3.4 (95% CI: 0.9–6.0; *p*=0.008) points higher on the CRIES-13 as compared with those where Criterion A1 was not met, whereas when Criterion A2 was met, children scored 11.9 (95% CI: 9.6–14.1; *p*<0.001) points higher as compared with those not meeting Criterion A2. The interaction term (A1*A2) was not significant and therefore not included in the linear regression analyses. The results are shown in Table 5.

**Table 5:** Relative strength and odds ratios of Criteria A1 and A2 in the prediction of posttraumatic stress

	Total score CRIES-13 (*β*)	Probable PTSD (odds ratio)
Criterion A1	3.4*	1.7*
Criterion A2	11.9**	8.9**

* *p*<0.05

** *p*<0.001.

Subsequently, a multiple logistic regression analysis was performed to evaluate the contribution of Criteria A1 and A2 to the prediction of probable PTSD. Criteria A1 and A2 explained 23% of the variance (Nagelkerke R^2^). When Criterion A2 was met, a child was nine times more likely to develop probable PTSD (OR 8.9; 95% CI: 4.8–16.5; *p*<0.001) as compared with those not meeting Criterion A2. When Criterion A1 was met a child was only two times more likely to develop probable PTSD (OR 1.7; 95% CI: 1.0–2.7; *p*=0.038) as compared with those where Criterion A1 was not met. The interaction term (A1*A2) was not significant and therefore not included in the logistic regression analyses. The results are shown in Table 5.

## Discussion

Findings of this study indicate that the combination of DSM-IV Criteria A1 and A2 make a significant contribution to the prediction of the posttraumatic stress response in children. However, the contribution of Criterion A2 is of greater importance than the contribution of Criterion A1. Children who met Criterion A2 reported significantly higher levels of posttraumatic stress and were nine times more likely to develop probable PTSD than children who did not meet Criterion A2. When Criterion A1 was met a child was only two times more likely to develop probable PTSD as compared with those where Criterion A1 was not met. Remarkably, these results are in contrast to most adult studies where it was found that Criterion A2 was of limited value (Adler et al., 2008; Bedard-Gilligan & Zoellner, 2008; Friedman et al., 2011). It might be the case that the subjective experience of an event plays a more prominent role in children.

The probability that someone who experienced a traumatic event will develop PTSD is known as the positive predictive value. Although children exposed to traumatic events are substantially at risk for developing PTSD, not all traumatic events result in PTSD. For this reason, we should not expect high positive predictive value from Criterion A. However, Weathers and Keane (2007) argue that as Criterion A is the initial requirement for the diagnosis PTSD, it should not rule out anyone who is clearly symptomatic and impaired (high sensitivity). Results from this study showed that almost all children with probable PTSD met Criterion A2. However, approximately 62% of the children with probable PTSD did not fulfill Criterion A1. This suggests that children may develop symptoms of posttraumatic stress regularly in the absence of Criterion A1. Similar results were found by others (Boals & Schuettler, 2009; Bodkin, Pope, Detke, & Hudson, 2007; Copeland, Keeler, Angold, & Costello, 2010; Gold, Marx, Soler-Baillo, & Sloan, 2005; Van Hooff et al., 2009). This finding is essential because it implies that research on PTSD should not be restricted to traumatic events as defined by Criterion A1.

The remarkably high negative predictive value of Criterion A2 indicates that if a child does not have a subjective reaction (Criterion A2) during an event it is unlikely that he or she will develop PTSD. These findings are in line with previous research in adults where the negative predictive value of Criterion A2 was emphasized (Bedard-Gilligan & Zoellner, 2008; Breslau & Kessler, 2001; Karam et al., 2010; Kilpatrick, Resnick, & Acierno, 2009). It suggests that Criterion A2 may be useful in a mass screening to filter out those children who are not at risk of developing PTSD.

### Limitations

First, data concerning Criteria A1 and A2 were based on retrospective subjective reports. Retrospective recall of adverse experiences and the person's response during or shortly after the event might be influenced by the presence of posttraumatic stress symptoms at the time of recall (Friedman et al., 2011). Furthermore, feelings of fear, helplessness, and horror are difficult to assess in children (especially younger children). Therefore, for further research it is recommended to include parents for reports on the observed reactions of the child and the duration and intensity of the symptoms.

Second, the posttraumatic stress response was assessed using a self-report measure, where no formal PTSD diagnosis was made. Duration (Criterion E) and subjective impairment in social, occupational, or other important areas of functioning (Criterion F) were not taken into account. It might be possible that children with “probable PTSD” according to the scores on the CRIES-13 did not meet other diagnostic criteria for a formal PTSD diagnosis. Standardized clinical interviews to assess a formal diagnosis of PTSD would have strengthened our results. However, the self-report measure used in this study (CRIES-13) has been found to be an accurate predictor for a PTSD diagnosis and has been widely used to assess the posttraumatic stress response and to screen children for PTSD (Perrin et al., 2005; Verlinden et al., 2013). Furthermore, the additional criterion of one positive score in each of the symptom clusters was added to strengthen our results.

Third, we have not assessed a complete trauma history due to time limitations. Therefore, we could not control for prior trauma. However, children were explicitly instructed to complete the questionnaire in reference to the described event.

Finally, the study was a sample of 533 school children, aged 8–18 years. However, most of these children are in secondary school and therefore caution should be taken when generalizing these findings to young children.

## Conclusion

Findings of this study raise fundamental questions about the value of the current Criterion A1. By using Criterion A1 as a threshold or “gatekeeper” to the diagnosis of PTSD, children with severe symptoms of posttraumatic stress who had experiences that do not fulfill Criterion A1, may not be eligible for treatment. Although the events that do not meet Criterion A1 may give rise to an adjustment disorder, these children may be excluded from receiving proper trauma-focused treatment. Furthermore, this study emphasizes the significant contribution of Criterion A2 to the prediction of the posttraumatic stress response in children. In accordance with Boals and Schuettler (2009), our findings suggest that it is not the nature of an event but rather the subjective experience that makes a life event traumatic. Nevertheless, a specific event is a necessary condition for the diagnosis of PTSD, but the type of event could be less objectively traumatic yet more subjectively traumatic for children. In other words, if a child has experienced an event that does not fully met Criterion A1, but subjectively experienced the event as traumatic (Criterion A2), we suggest that he or she could still be diagnosed with PTSD. What really matters is whether the existing posttraumatic stress experienced by a child causes significant impairment in social, occupational, or other important areas of functioning. These children might benefit from trauma-focused treatment, regardless of whether the event they have experienced met Criterion A1 or not. These findings have important implications for the development of an accurate PTSD definition for children in the upcoming DSM-5.
